# Identification and biochemical characterization of a novel porcine circovirus associated with porcine respiratory and diarrheal diseases

**DOI:** 10.1128/spectrum.02299-25

**Published:** 2025-10-08

**Authors:** Xianhui Liu, Lin Wang, Xinming Zhang, Yilong Liu, Yixuan Li, Zan Li, Zhi Geng, Leyi Zhang, Yanling Liu, Pengshuai Liang, Yuhui Dong, Zheng Xu, Heng Zhang, Changxu Song

**Affiliations:** 1 State Key Laboratory of Swine and Poultry Breeding Industry, National Engineering Research Center for Breeding Swine Industry, College of Animal Science, South China Agricultural University 12526, Guangzhou, China; 2 Beijing Synchrotron Radiation Facility, Institute of High Energy Physics, Chinese Academy of Sciences, Beijing, China; Public Health Agency of Canada, Winnipeg, Manitoba, Canada

**Keywords:** CRESS DNA viruses, circoviruses, virus-like particles, pathogenesis

## Abstract

Eukaryotic circular Rep encoding single-stranded DNA (CRESS DNA) viruses exhibit high genomic diversity. Four species of circoviruses within the genus *Circovirus* have been identified in pigs, including the non-pathogenic porcine circovirus type 1 (PCV1), and the pathogenic PCV2, PCV3, and PCV4. In this study, a novel single circular DNA virus was identified in pigs suffering from respiratory, diarrheal, and reproductive failure diseases. The viral Rep and Cap showed low amino acid sequence identities to the four PCV species, with the highest identity of 26.2% to PCV2 Rep and 20.8% to PCV4 Cap. The classification of CRESS DNA viruses primarily relies on Rep, and biochemical analyses of the viral Rep confirm its classification as a CRESS DNA virus. Thus, it was tentatively named porcine circovirus type 5 (PCV5). This tentative designation reflects its host and genomic organization; however, phylogenetic analysis indicates that it clusters outside the family *Circoviridae* and is most closely related to the fur seal faces-associated circular DNA virus (FSfaCV). The positive detection rate for PCV5 in tested samples was 54.4% (294 out of 540), with 24.3% (17 out of 70) of pig farms in China testing positive for the virus. A close correlation between the copy number of PCV5 and the severity of infectious diseases was observed. Moreover, a PCV5 virus-like particles (VLPs)-based enzyme-linked immunosorbent assay, which was developed, also elucidated that PCV5 has been circulating. We subsequently established an *in vitro* culture system for PCV5, successfully purifying the virus. Morphological identification revealed that the purified PCV5 has a diameter of approximately 20 nm, and the recombinant PCV5 Cap could assemble into VLPs similar to purified PCV5. These results indicate that PCV5, associated with porcine circovirus-associated disease, commonly circulates within Chinese swine.

**IMPORTANCE:**

The single-stranded circular DNA virus, tentatively named porcine circovirus type 5 (PCV5), belongs to the circular Rep encoding single-stranded DNA (CRESS DNA) viruses. Notably, PCV5 is not classified within the same family as the previously reported PCV1–PCV4. PCV5, which was associated with porcine circovirus associated-disease, is widely prevalent in Southern China. PCV5 is characterized by a larger genome (2,904 nt), along with longer Cap (1,182 nt) and Rep (1,056 nt) regions, as well as an extended stem-loop structure (stem: 9 nt and loop: 17 nt). Importantly, we subsequently established an *in vitro* culture system for PCV5, successfully purifying the virus. Morphological identification revealed that the purified PCV5 has a diameter of approximately 20 nm. Additionally, a variant of the PCV5 Cap, lacking the N-terminal 63 residues, was expressed in *Escherichia coli*, and purified Cap could assemble into virus-like particles similar to purified PCV5. These results hold significant implications for the study of CRESS DNA viruses. The emergence of PCV5 warrants further study.

## INTRODUCTION

Eukaryotic circular Rep encoding single-stranded DNA (CRESS DNA) viruses, which encode replication-related proteins (Rep) and infect eukaryotic organisms, possess the characteristics of small viral genomes, diverse host ranges, high prevalence, and strong rolling circle replication affinity. CRESS DNA viruses infect a wide array of different eukaryotes, such as plants, animals, humans, and fungi, and possess far-reaching and important implications in virology (1, 2). Those viruses are small non-enveloped DNA viruses with a single-stranded circular genome, primarily encoding two proteins: capsid-related protein (Cap) and replication-associated protein (Rep) (1, 2). The Cap is the sole protein component of the virus particles and plays a crucial role in the entire virus replication cycle, participating in virus attachment, cell entry, genome uncoating, and the packaging of newly formed virus particles (1, 3). The Rep protein facilitates the recognition of replication initiation within the viral genome sequence and possesses endonuclease activity, which cleaves the circular DNA and initiates rolling circle replication (1, 3, 4).

According to the International Committee on Taxonomy of Viruses, CRESS DNA viruses, the classification of which is based on the Rep amino acid, currently include seven family members, namely *Circoviridae*, *Nanoviridae*, *Smacoviridae*, *Genomoviridae*, *Bacilladnaviridae*, *Geminiviridae*, and *Kirkoviridae* (1, 2). With the rapid advancement and application of new technologies such as metagenomic sequencing, novel types of CRESS DNA viruses are continuously being discovered and classified. Among the extensively studied CRESS DNA viruses, the family *Circoviridae* includes two genera: Cyclovirus and Circovirus (3), which are the pathogens causing porcine circovirus-associated diseases (PCVADs) (5) and beak and feather disease in birds (6), respectively. Notably, porcine circovirus type 2 (PCV2) and beak and feather disease virus (BFDV) are the most thoroughly researched CRESS DNA viruses to date (3, 5, 7). These viruses identified in pigs include PCV1 (5), PCV2 (5), PCV3 (8), PCV4 (9), and porcine circovirus-like virus (PCLV) (10, 11). PCV2 has been associated with clinical diseases in pig farms known as PCVAD, causing substantial economic losses. At present, PCV2 and PCV3 are prevalent in the global pig industry (5, 12).

In recent years, several new human CRESS DNA viruses have been discovered and studied. Researchers used metagenomic next-generation sequencing to identify an unknown species of circovirus, designated human circovirus 1 (HCirV-1), from a liver biopsy sample (13). Additionally, the researchers identified a novel circovirus, human-associated circovirus 2 (HuCV2) from the blood of two intravenous drug users in China (14). Furthermore, the researchers reported a new Circovirus in a patient in France who had acute hepatitis of unknown origin using means of routine shotgun metagenomics (15).

Infections of unknown origin must be diagnosed promptly to ensure that appropriate measures are implemented in a timely manner to prevent the spread of potentially harmful pathogens and to facilitate effective treatment (15). Significant advancements have been made in next-generation sequencing metagenomic sequencing of viral communities, which has been extensively utilized for monitoring unknown diseases in humans, animals, plants, and other organisms (1, 13, 15). Additionally, designing universal primers based on uploaded metagenomic sequences in GenBank, along with existing research sequences, provides an efficient and cost-effective method for monitoring homologous viruses within a specific family or genus.

In this study, we identified a novel CRESS DNA virus, tentatively named porcine circovirus type 5 (PCV5). This tentative designation reflects its host and genomic organization; however, phylogenetic analysis indicates that it clusters outside the family *Circoviridae* and is most closely related to the fur seal faces-associated circular DNA virus (FSfaCV). PCV5 is associated with respiratory and diarrheal diseases in piglets, as well as reproductive failure diseases.

## RESULTS

### Epidemiologic investigation of a novel single-stranded circular DNA virus

**Fig 1:**
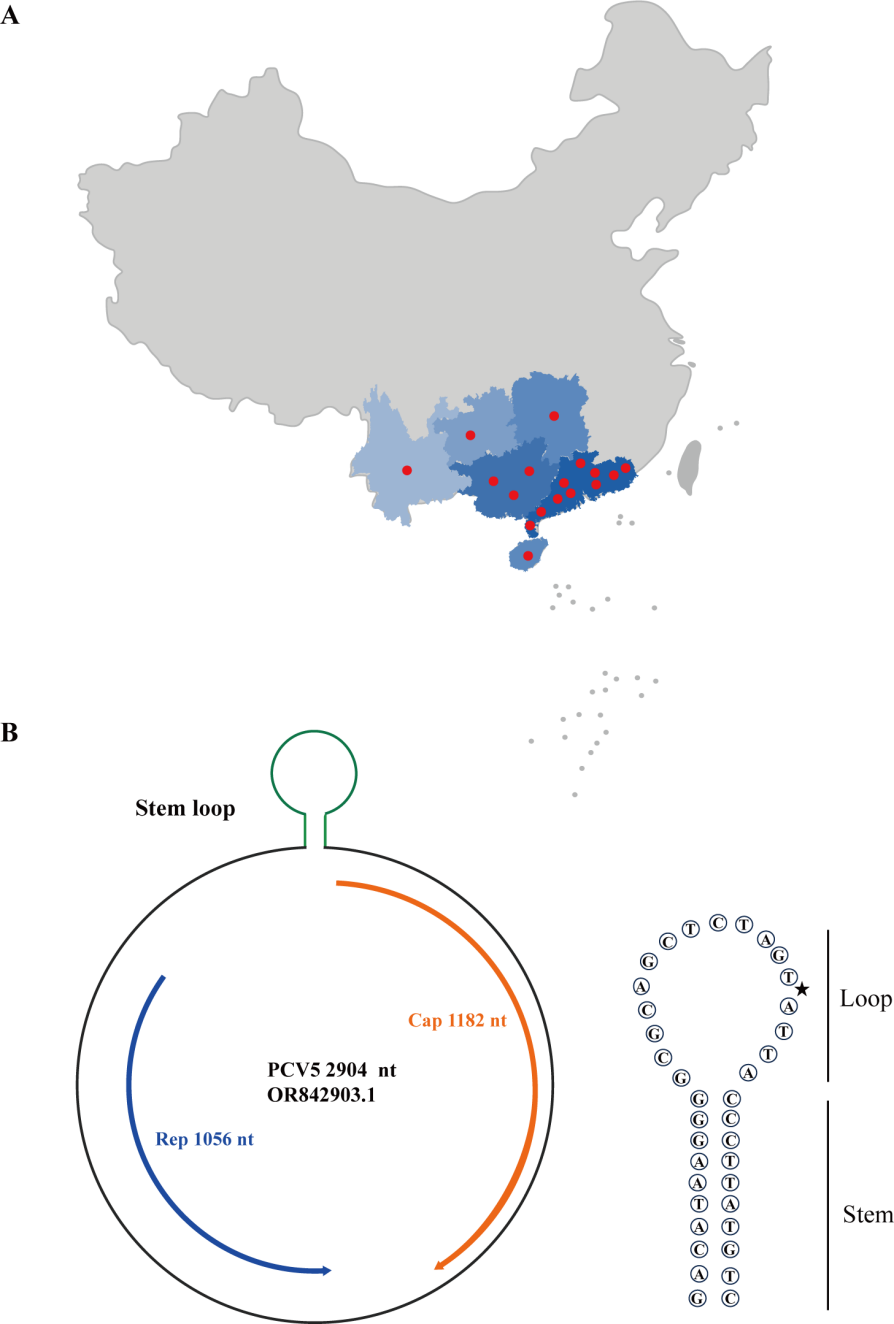
Geographic distribution and genome organization of the PCV5. (**A**) Areas where surveillance of PCV5 was carried out are shown in blue. Red circles indicate the locations of farms with confirmed PCV5 infection. A total of 17 pig farms in Guangxi, Guangdong, Hunan, Yunnan, and Hainan provinces were PCV5 positive. (**B**) PCV5 has a single-stranded circular genome (2,904 nt): the Cap (1,182 nt), the Rep (1,056 nt), and a stem ring (stem: 9 nt and loop: 17 nt).

A novel single-stranded circular DNA virus, tentatively named PCV5, was identified by designing universal primers based on uploaded metagenomic sequences in GenBank, along with existing research sequences. From October 2021 to June 2023, we identified 17 swine farms with pigs exhibiting respiratory, diarrheal, and reproductive failure diseases that were infected with PCV5. Pigs were classified as having PCV5 infection if PCV5 DNA was detected in clinical samples. This study found that 24.3% (17/70) of the pig farms tested positive for PCV5, indicating that PCV5 is widely prevalent in south China (Fig. 1A). However, this does not provide information on the transmission dynamics and epidemiology of PCV5. Further research on the prevalence of PCV5 in different areas and seasons is necessary.

### Sequencing and genetic analysis of the PCV5

Following PCR and Sanger sequencing of the resulting amplicons, we assembled a 2,904 nt circular genome from the intestinal homogenate of a piglet (Fig. 1B). The PCV5 genome analysis identified two open reading frames (ORFs) that encode proteins exceeding 300 amino acids (aa), with two ORFs showing significant homology to Rep and Cap of circoviruses by BLASTP, structural prediction, and comparison. The Cap is 1,182 nt, the Rep is 1,056 nt, and a stem-loop is 26 nt in length (stem: 9 nt and loop: 17 nt), which is significantly longer than PCV1, PCV2, PCV3, PCV4, and several human circoviruses (16) (Fig. 1B; Table S1 ).

### Genetic relationship of PCV5 strains to other CRESS DNA viruses

**Fig 2:**
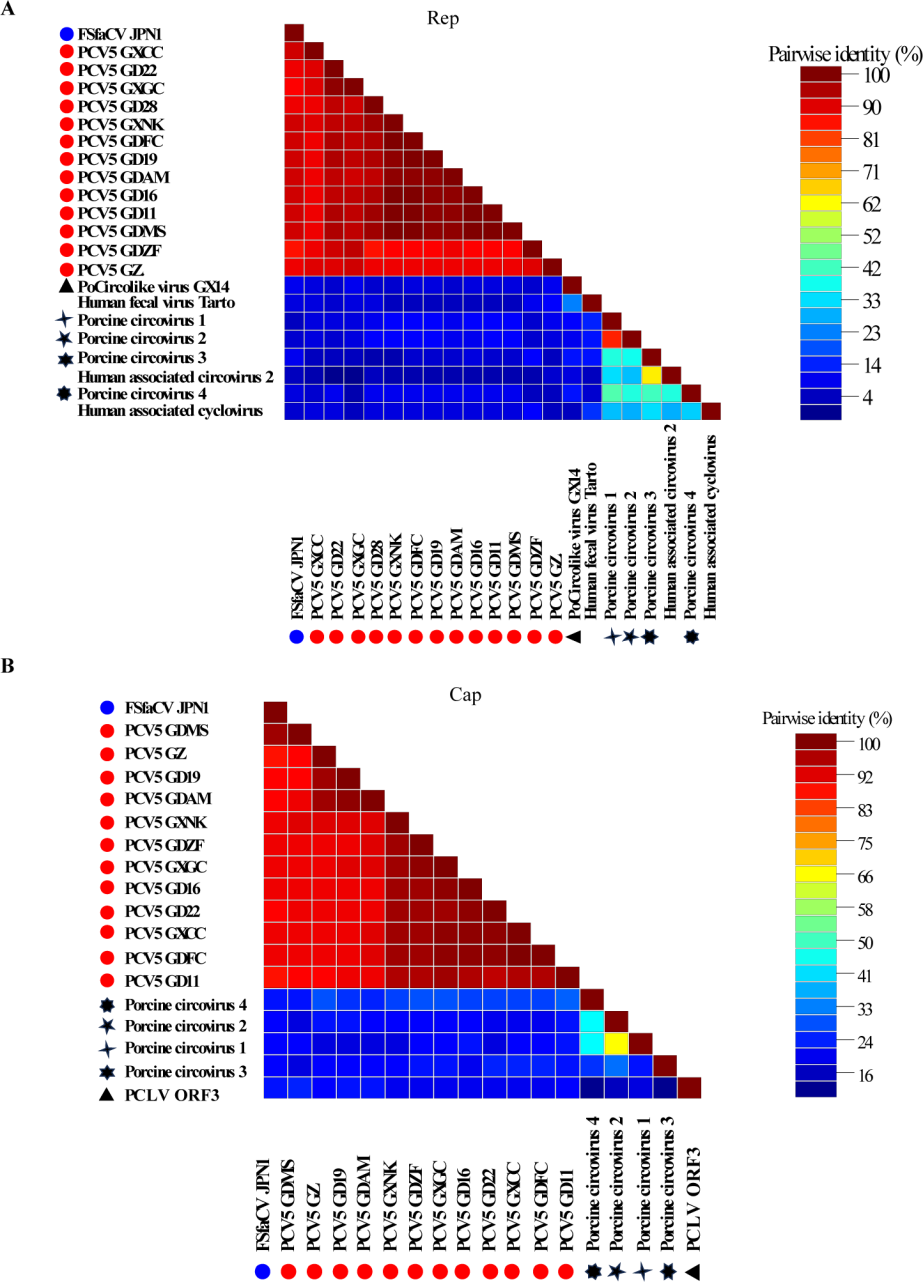
Genetic relationship of PCV5 strains to PCV1, PCV2, PCV3, PCV4, PCLV, and several human circoviruses. Pairwise identities of the Rep (**A**) and Cap (**B**) amino acid of 12 PCV5 strains to other circoviruses. The black star indicates PCV1, PCV2, PCV3, PCV4, and PCLV, and the blue circle represents the FSfaCV (accession KF246569.1) strain.

The amino acid sequence of PCV5 Rep was significantly different from those of PCV1, PCV2, PCV3, PCV4, PCLV, and several human CRESS DNA virus Rep, and the similarity was less than 26.2% (Fig. 2A). The similarity of Rep amino acid sequence between the 12 PCV5 strains ranged from 87.7% to 99.7%, and the similarity between these strains and the FSfaCV (accession KF246569.1) strain ranged from 85.8% to 92.6% (Fig. 2A). The novel circovirus identified in this paper is highly homologous to FSfaCV. The study of FSfaCV is limited, and its prevalence is unknown. This virus is closely related to PCVAD and is widely prevalent in pigs, which indicates that it may be an important pathogen, and for the convenience of subsequent studies, it is temporarily named PCV5. The amino acid of PCV5 Cap sequence was significantly different from those of PCV1, PCV2, PCV3, PCV4, PCLV, and several human CRESS DNA virus Cap, with a similarity of less than 20.8% (Fig. 2B). And the amino acid similarity of Cap between these 12 PCV5 strains and the FSfaCV strain ranges from 89.1% to 99.2% (Fig. 2B). These results indicated that PCV5 is a novel single circular DNA virus.

### Biochemical characteristics of PCV5 Rep

**Fig 3:**
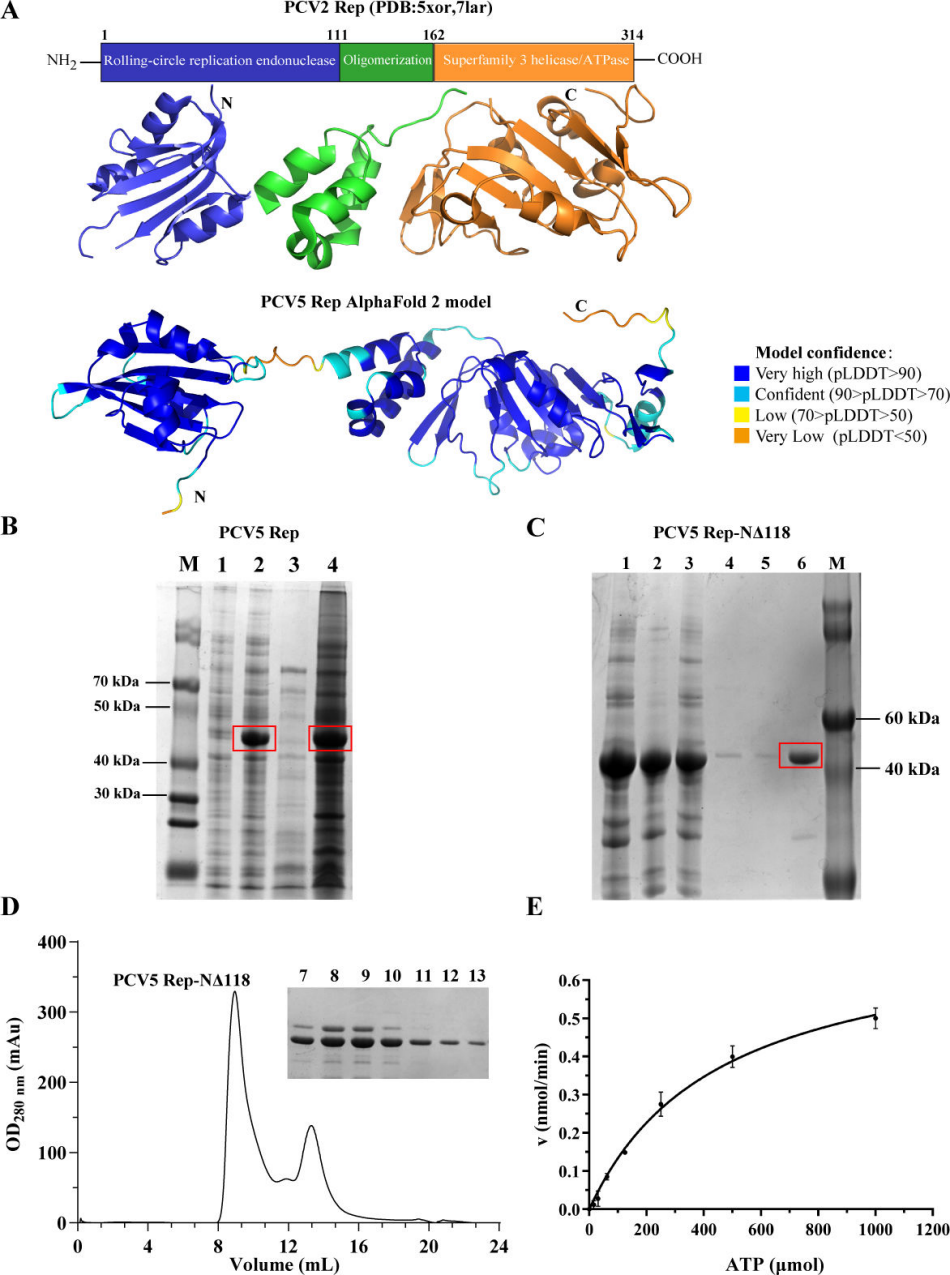
Determination of soluble expression and ATPase activity of PCV5 Rep. (**A**) Comparison of the predicted PCV5 Rep and PCV2 Rep structure. In the predicted PCV5 Cap structure, the colors blue, cyan, yellow, and orange mean very high, confident, low, and very low predicted local distance difference test (pLDDT) score, respectively. The pLDDT (between 0 and 100) means the distribution of per-residue confidence score. N indicates the N-terminal of the protein, and C indicates the C-terminal of the protein. (**B**) Electrophoresis results of PCV5 full-length Rep. M is a molecular weight marker. Lane 1 is the pre-induction sample. Lane 2 is the sample after induction. Lane 3 is the supernatant sample after induction. Lane 4 induced precipitation samples. (**C**) PCV5 Rep NΔ118 (the oligomerization region and C-terminal of PCV5 Rep) can be stably expressed in prokaryotic system on the PGEX-GST plasmid, and M is the molecular weight marker. Lane 1 is the pre-induction sample. Lane 2 is induced precipitation. Lane 3 is the supernatant sample through the nickel column flow sample. Lane 4 is 30 mM imidazole. Lane 5 is 50 mM imidazole. Lane 6 is 300 mM imidazole elution. (**D**) The protein concentration after the result of molecular sieve and the result of electrophoresis. (**E**) ATPase activity of PCV5 Rep NΔ118 was detected.

The classification of CRESS DNA viruses is primarily based on Rep. We subsequently investigated the biochemical functions of PCV5 Rep. It has been mentioned above that the amino acid sequence of the Rep and Cap of PCV5 is significantly different from those of PCV1, PCV2, PCV3, PCV4, PCLV, and several human CRESS DNA viruses. Subsequently, we predicted the Rep and Cap structures of PCV5 using Alpha-Fold2. The structure of PCV5 Rep is highly similar to the structure of PCV2 Rep (PDB: 5XOR, 7IAR); both possess the classic three domains: N-terminal endonuclease domain, oligomerization domain, and superfamily helicase/ATPase domain (Fig. 3A). The above results indicated that even with low sequence identities, the Rep structures among CRESS DNA viruses are highly conserved.

Then, we attempt to obtain the full-length recombination PCV5 Rep. However, the full-length Rep was expressed in *Escherichia coli* as an inclusion body form (Fig. 3B). According to the AF2 model, we truncated the N-terminal endonuclease domain of PCV5 Rep (termed as PCV5 Rep NΔ118), and the PCV5 Rep NΔ118 was soluble (Fig. 3C). Size-exclusion chromatography showed that PCV5 Rep NΔ118 exists as an oligomeric form in solution (Fig. 3D). In order to investigate whether PCV5 Rep NΔ118 exhibits ATPase activity, the enzymatic kinetics of purified PCV5 Rep NΔ118 were studied using the malachite green phosphate colorimetric assay. The enzymatic kinetics data were well fitted to the Michaelis-Menten equation (Fig. 3E). Additionally, PCV5 Rep was mainly located in the cytoplasm, although a small part of Rep could enter the nucleus (Fig. S2A). Thus, the expression of purified PCV5 Rep NΔ118 exhibits ATPase activity. These biochemical results further indicate that PCV5 Rep was similar to the classical Rep of CRESS DNA viruses, and PCV5 can be classified into CRESS DNA viruses.

### Phylogenetic analysis based on Rep

**Fig 4:**
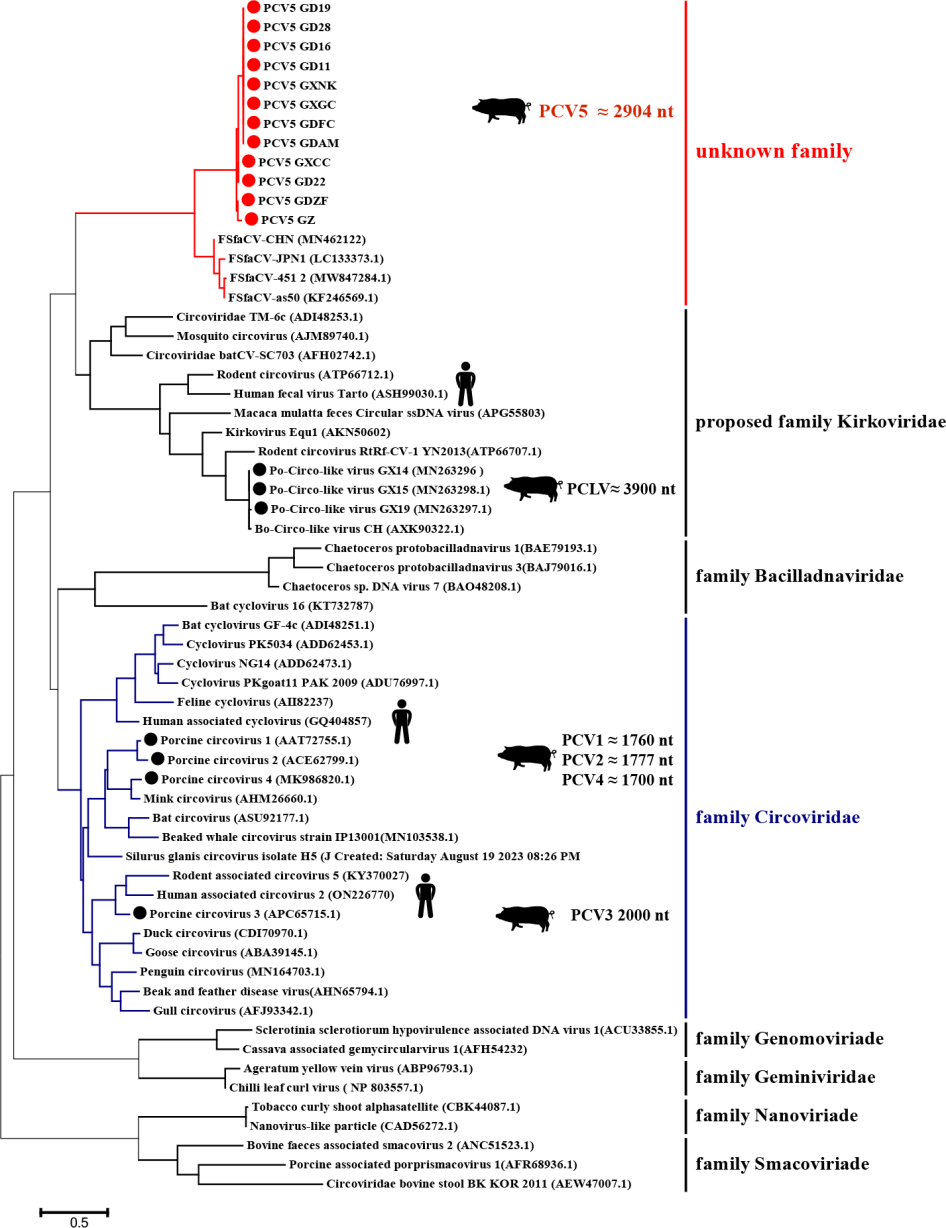
A phylogenetic tree was constructed based on amino acid sequence of Rep protein of the CRESS DNA viruses. The coding regions for the Rep protein were aligned and analyzed using means of the neighbor-joining method with Poisson correction and complete deletion of gaps. Bootstrap testing (1,000 replicates) was used, and the bootstrap values were indicated. The PCV5 strains in the present study are marked with red circles. Black circles indicate five porcine circoviruses: PCV1, PCV2, PCV3, PCV4, and PCLV.

To further investigate the evolutionary relationship of PCV5 to other CRESS DNA viruses, we analyzed genome sequences from 49 representatives of the different family and 12 amino acid sequences of PCV5 Rep. The phylogenetic analysis (Fig. 4) indicates that PCV5 is most closely related to the FSfaCV (accession KF246569.1). The phylogenetic tree (Fig. 4) also suggests that PCV5 is genetically distinct from PCV1, PCV2, PCV3, PCV4, PCLV, and several human CRESS DNA viruses, indicating that PCV5 does not belong to the known family. Furthermore, PCV5 exhibits significant differences in genome length compared to PCV1, PCV2, PCV3, PCV4, PCLV, and several human CRESS DNA viruses. These results indicated that PCV5 is a novel CRESS DNA virus.

### Histological lesions associated with the presence of PCV5

To confirm the presence of PCV5 in porcine samples, a qPCR assay was developed to detect the PCV5 Rep gene (Fig. S2 to S4), which exhibited good specificity and repeatability. The tissue homogenate samples from 12 piglets in Guangxi were strongly positive for PCV5, with cycle threshold (CT) values between 11.1 and 34 (Table 1). The virus CT values of 12 pigs were closely correlated with their clinical symptoms. When viral DNA levels were low, higher concentrations of PCV5 DNA were found in intestinal tissue, intestinal lymph nodes, and feces. However, as clinical symptoms of piglets become more severe, elevated levels of viral DNA were also detected in other organs. It was particularly notable to find that the virus can also cross the blood-brain barrier, as high levels of viral DNA were identified in brain samples. In addition, PCV5 DNA was detected in stillbirth piglets from other farms with outbreaks of reproductive disorders (data are not shown). These results demonstrate that PCV5 was detected in multiple organs and its association with PCVAD.

**TABLE 1:** CT values of PCV5 virus in the organs of 12 piglets^*a*^

	Pig number											
	01	02	03	04	05	06	07	08	09	10	11	12
Organ												
Heart	31.7	–	38.1	34.1	–	–	23.8	32.0	34.7	36.3	30.3	30.5
Liver	33.0	34.2	36.5	35.1	36.2	31.7	25.5	29.8	–	37.4	28.3	33.2
Spleen	33.1	35.5	–	34.5	35.5	33.2	22.3	32.2	36.8	35.4	32.9	32.6
Lung	31.8	34.1	–	35.2	34.0	31.8	25.4	29.4	34.0	35.3	29.4	31.2
Kidney	34.5	–	–	33.5	32.3	30.9	23.4	31.4	33.4	34.5	30.4	32.6
Brain	30.8	33.7	34.5	34.6	33.3	30.7	25.1	28.4	33.0	32.3	27.5	29.6
Duodenum	33.1	35.7	34.8	32.9	33.1	31.3	22.2	34.9	29.3	27.2	25.0	26.5
Jejunum	32.6	33.2	36.9	34.8	–	29.3	24.1	34.6	31.9	35.4	29.1	30.9
Ileum	28.7	34.0	37.9	33.3	35.8	33.7	26.0	29.9	32.4	36.4	25.2	30.6
Cecum	37.2	35.6	30.1	31.9	33.0	23.8	17.3	30.8	28.9	26.5	23.6	32.0
Colon	33.5	33.9	28.9	28.4	23.9	18.9	13.4	30.7	21.4	24.5	27.4	30.9
Rectum	32.9	35.1	30.7	23.2	24.3	18.3	17.3	29.6	24.9	25.7	21.9	31.4
Submaxillary lymph nodes	31.3	35.5	–	30.4	30.0	36.4	24.3	28.8	38.3	32.0	26.4	28.6
Bronchopulmonary hilar lymph nodes	29.1	34.3	36.8	34.6	33.8	34.4	26.7	33.9	–	32.3	29.6	23.5
Mesenteric lymph nodes	32.9	–	35.4	30.2	34.9	–	19.3	34.5	35.3	28.4	26.0	21.3
Inguinal lymph nodes	31.3	32.7	–	30.8	31.1	29.8	19.8	31.3	30.2	30.1	29.2	22.5
Tonsil	31.5	35.0	32.3	34.3	34.2	31.9	21.5	30.4	29.2	32.4	25.4	24.2
Feces	24.2	32.6	31.1	16.9	11.4	34.2	11.1	30.6	28.3	32.7	21.8	31.3

^*a*^ – indicates that no CT value was detected.

**Fig 5:**
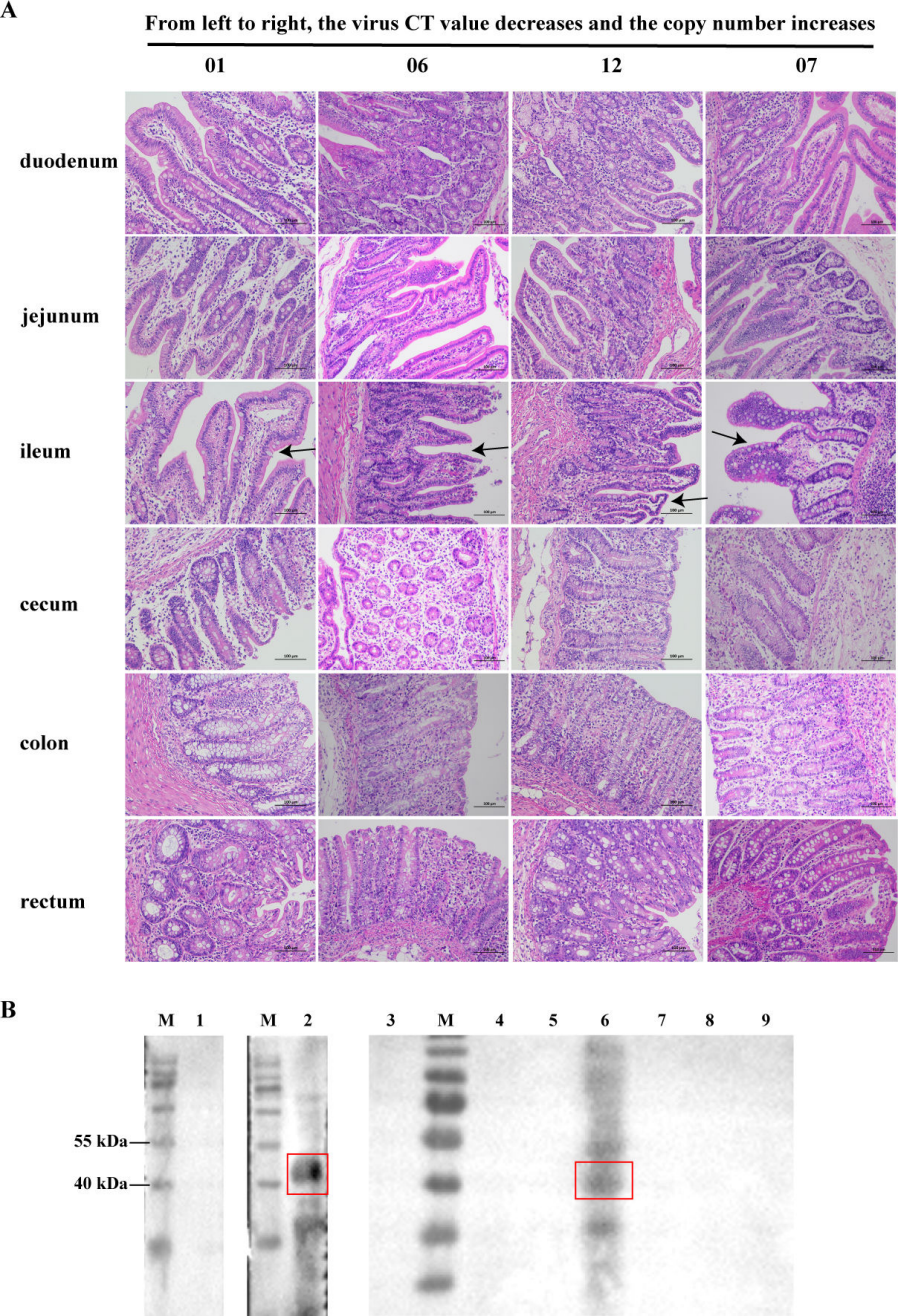
Histologic lesions of tissue of swine infected with PCV5. (**A**) Histologic section of duodenum, jejunum, ileum, cecum, colon, rectum, and lung from PCV5-positive piglets (01, 06, 12, and 07) stained with HE. Four piglets were selected, numbered 01, 06, 12, and 07, and the CT value of the PCV5 gradually decreased. (**B**). The prepared polyclonal antibody was tested by Western blotting to detect PCV5 Cap in the positive tissue, with M representing marker, 1 representing negative control, 2 representing positive control, and 3–9 representing clinical samples of PCV5-positive pigs (number 07): duodenum, jejunum, ileum, cecum, colon, rectum, and lung.

Then, four piglets, numbered 01, 06, 12, and 07, were selected and stained with hematoxylin and eosin (HE). The PCV5 CT value of the above four piglets gradually decreased (Table 1). The pathological examination of the four piglets revealed that the organ lesions were associated with the copies of PCV5 DNA (Fig. 5A)**,** and the pathological injury of the ileum was serious in the four piglets. These results suggest a close correlation between the copy number of PCV5 and the severity of infectious diseases. Western blotting showed that the prepared polyclonal antibody can detect the viral protein in the colon sample of porcine (number 07) infected with PCV5 (Fig. 5B). These results also further confirm the association between PCV5 and histological lesions of piglets.

### Virus isolation and visualization

**Fig 6:**
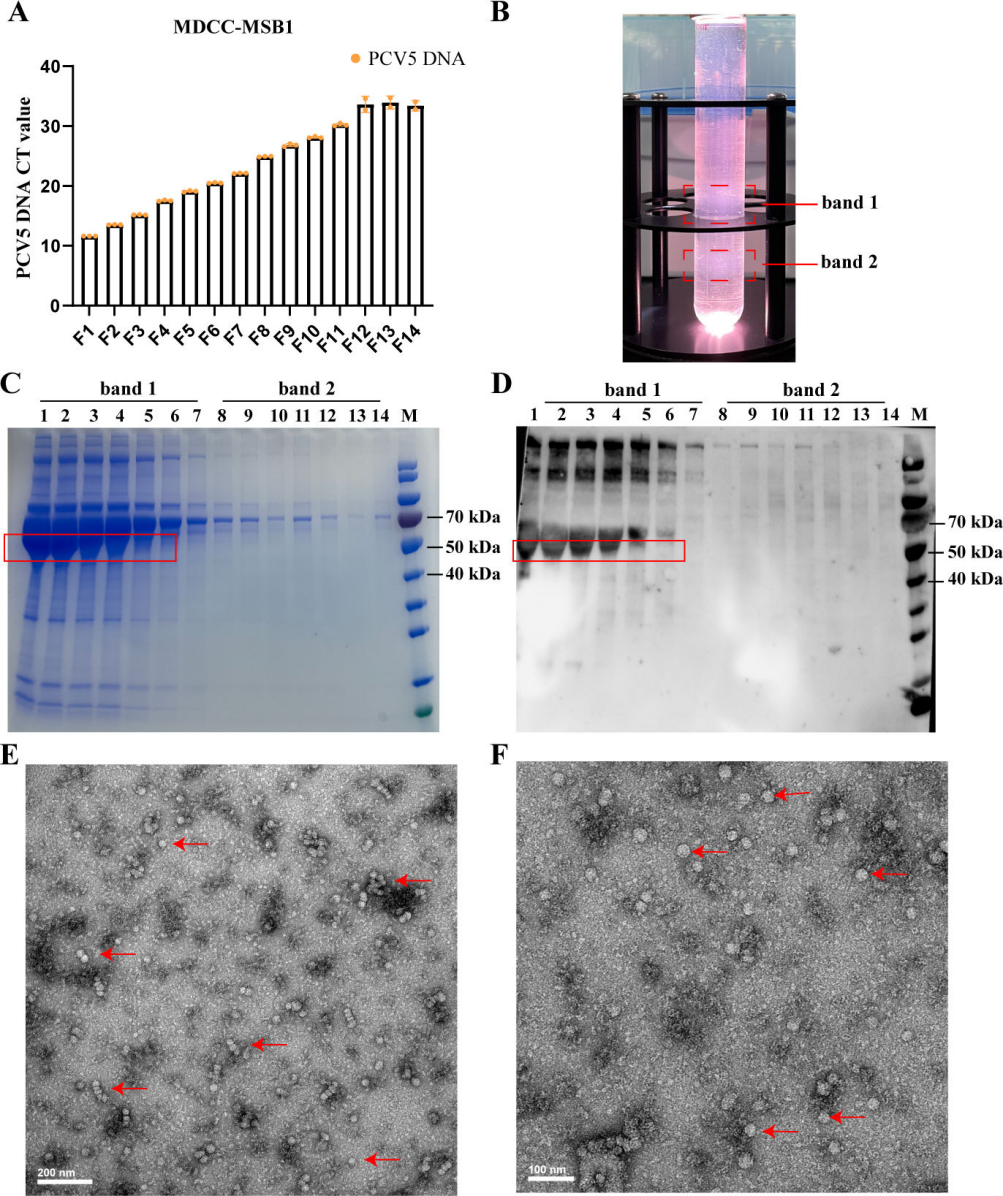
*In vitro* propagation and identification of PCV5 virus. (**A**) Culture of PCV5 on MDCC-MSB1 cells. (**B**) Purification of PCV5 by discontinuous density ultracentrifugation using the 30%–50% (wt/vol) sucrose density gradient. (**C**) SDS-PAGE analysis of fractions from discontinuous sucrose gradient centrifugation. Lanes 1–7 represent the band 1 (200 μL from top to bottom), and Lanes 8–14 represent the band 2 (200 μL from top to bottom). (**D**) Western blot analysis of fractions from discontinuous sucrose gradient centrifugation. Lanes 1–7 represent the band 1 (200 μL from top to bottom), and Lanes 8–14 represent the band 2 (200 μL from top to bottom). (**E**) Negative stain microscope detection of purified PCV5 virus. The scale bar is 200 nm. (**F**) Negative stain microscope detection of purified PCV5 virus. The scale bar is 100 nm. The red arrow represents the viral particle.

The PCV5 isolation from the intestinal tissue of piglets was performed in Marek’s disease lymphoma cell line (MDCC-MSB1). After serial generations of the blind passages, the PCV5 isolate was obtained in MDCC-MSB1 cells (Fig. 6A). MDCC-MSB1 cells infected with PCV5 showed no cytopathic effects (Fig. S5A). For the discontinuous sucrose gradient centrifugation, the cell supernatant containing PCV5 was purified with a discontinuous 30%–50% (wt/vol in phosphate-buffered saline [PBS]) sucrose gradient (Fig. 6B).

Then, the purified sample was examined. SDS PAGE indicates that PCV5 exists in the band 1 (Fig. 6C), and western blot also indicates that PCV5 was distributed in the band 1 using PCV5 Cap polyclonal antibody (Fig. 6D). Subsequently, the sample containing PCV5 was visualized by negative-stained electron microscopy. Many non-enveloped icosahedral particles of approximately 20 nm in diameter, morphologically with the characteristic features of the family *Circoviridae*, were observed (Fig. 6E and F). In addition, we also observed virions under electron microscopy, after the sample was purified using continuous 10%–50% (wt/vol in PBS) linear sucrose gradient centrifugation (Fig. S5B through E). These results confirmed that we successfully established an *in vitro* culture system for PCV5, and PCV5 was successfully purified. The morphological identification of the purified PCV5 revealed that PCV5 is similar to PCV2.

### Self-assembly of PCV5 virus-like particles and their application in PCV5 seroprevalence

**Fig 7:**
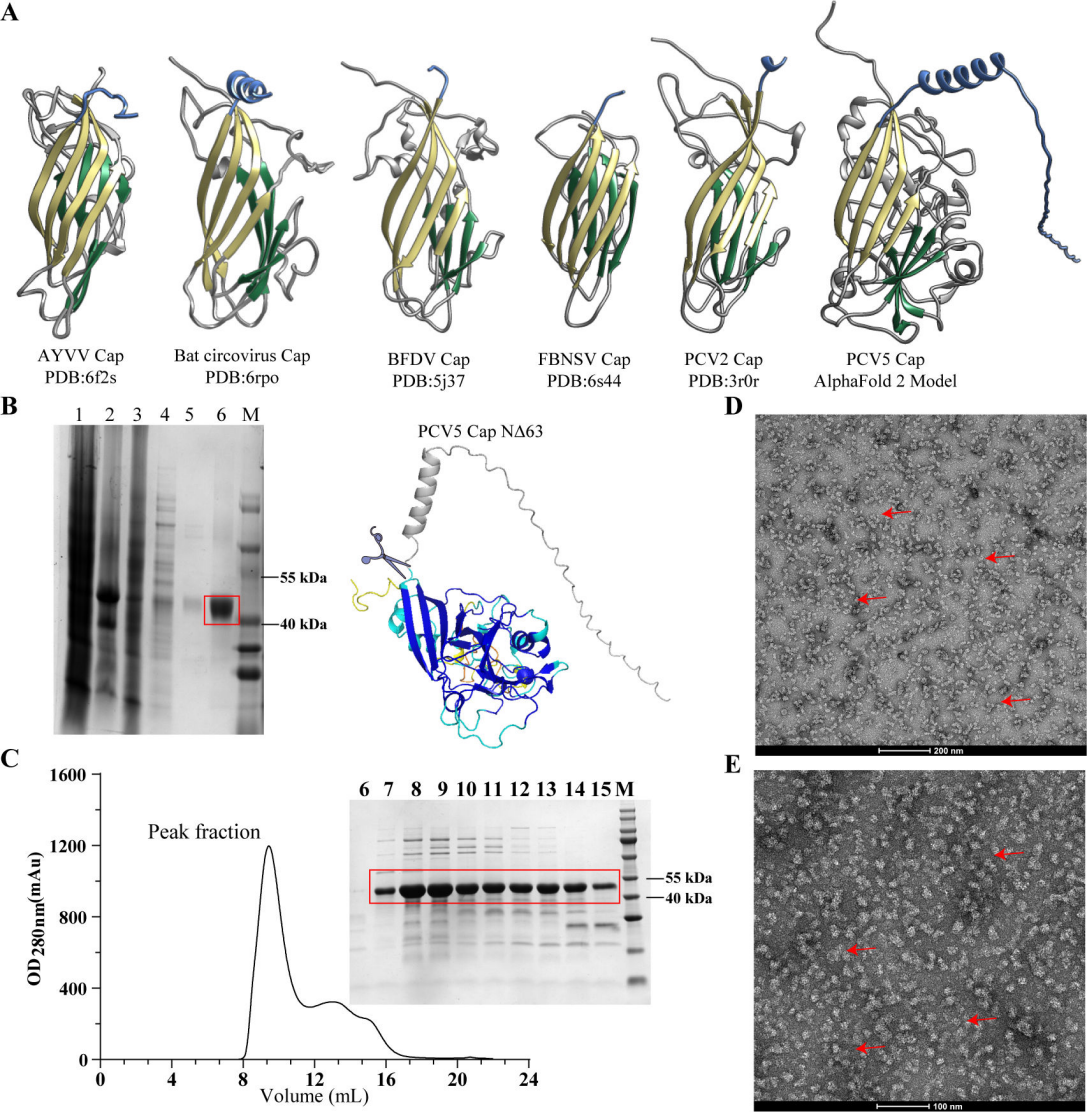
Expression, purification, and assembly of the PCV5 VLPs. (**A**) Structural comparison of predicted PCV5 Cap (by Alpha-Fold2) with known Cap proteins of CRESS DNA viruses. The Cap structures are from PCV2, BFDV, and bat circovirus, both of which belong to *Circoviridae* family. The Cap structure is from Faba bean necrotic stunt virus (FBNSV) belonging to *Nanoviridae* family. The Cap structure in blue is from Ageratum yellow vein virus (AYVV) Cap structure belonging to *Nanoviridae* family. These Cap structures in cornflower blue indicate N-terminal domain, and these Cap structures in khaki and sea green indicate the jelly roll domain, which consists of two β-sheets, each containing four β-strands connected by loop. (**B**) Purification of PCV5 Cap-NΔ63 by nickel column. M stands for marker, 1 indicates the supernatant after cracking, 2 indicates precipitation after cracking, 3 represents the flow fluid purified by nickel column, 4 indicates 30 mM imidazole, 5 indicates the cleaning of 50 mM imidazole, and 6 indicates 300 mM imidazole elution during nickel column purification. In the predicted PCV5 Cap structure, the colors blue, cyan, yellow, and orange mean very high, confident, low, and very low pLDDT score, respectively. The pLDDT (between 0 and 100) means the distribution of per-residue confidence score. (**C**) Purification of PCV5 Cap-NΔ63 by size-exclusion chromatography (Superose 6). (**D and E**) Transmission electron microscopy of PCV5 Cap VLPs. (**D**) The scale bar is 200 nm. (**E**) The scale bar is 100 nm. The red arrow represents the virus-like particles.

In order to obtain a substantial quantity of recombination Cap protein, we attempted to express PCV5 Cap in *E. coli*. However, we found that full-length Cap could not express in *E. coli*. We found the Cap structure of PCV5 is highly similar to the Cap structure of the published CRESS DNA virus (17–22), which is a classic jelly roll (Fig. 7A). The jelly roll domain consists of two β-sheets, each of which contains four β-strands connected by loop (23–25). The above results indicated that even with low sequence identities, the Cap structures among CRESS DNA viruses are highly conserved. Additionally, full-length PCV5 Cap exists in the cell nucleus (Fig. S2B). PCV5 Cap could be expressed in large quantities after the deletion of 63 amino acids at the N-terminal (Fig. 7B). Size-exclusion chromatography indicated that the truncated Cap exists as an oligomeric form in solution, and the molecular sieve peak was 8–9 mL (Fig. 7C). The purified PCV5 Cap was capable of self-assembly into virus-like particles (VLPs) (size: 17–22 nm), which was observed by transmission electron microscopy (Fig. 7D and E). Subsequently, PCV5 Cap virus-like particles with adjuvant were prepared into a vaccine and injected into rabbits to prepare polyclonal antibodies.

The prevalence of anti-PCV5 Cap antibodies in porcine serum samples was assessed using an enzyme-linked immunosorbent assay (ELISA) with PCV5 Cap VLPs. The externally purified PCV5 Cap VLPs exhibit characteristics such as uniformity and stability. This study established an indirect ELISA serological detection method utilizing PCV5 VLPs, which exhibited good specificity and repeatability (Tables S2 and S3). Anti-PCV5 Cap antibodies were detected in 524 (66.84%) of 784 pig serum samples collected from multiple regions (Table 2). Among the positive samples, 298 originated from Guangdong, 56 were from Guangxi, 78 were from Hunan, and 92 were from Yunnan (Table 3). The results of PCV5 seroprevalence revealed that PCV5 has been circulating in Southern China.

**TABLE 2:** Positive rate of PCV5 antibody in Chinese provinces

Location/provinces	Number of farms	Number of samples	Number of positive samples	Positive rate (%)
Guangdong	20	417	298	71.46
Guangxi	5	135	56	41.48
Hunan	4	113	78	69.02
Yunnan	3	119	92	77.31
Total	32	784	524	66.84

**TABLE 3:** Positive rate of PCV5 antibody in serum samples from pigs at different stages

Types of pigs	Number of samples	Number of positive samples	Positive rate (%)
Farrowing piglet	57	30	52.63
Nursery pig	94	48	51.06
Fattening pig	102	67	65.69
Sow	531	379	71.37
Total	784	524	66.84

## DISCUSSION

**Fig 8:**
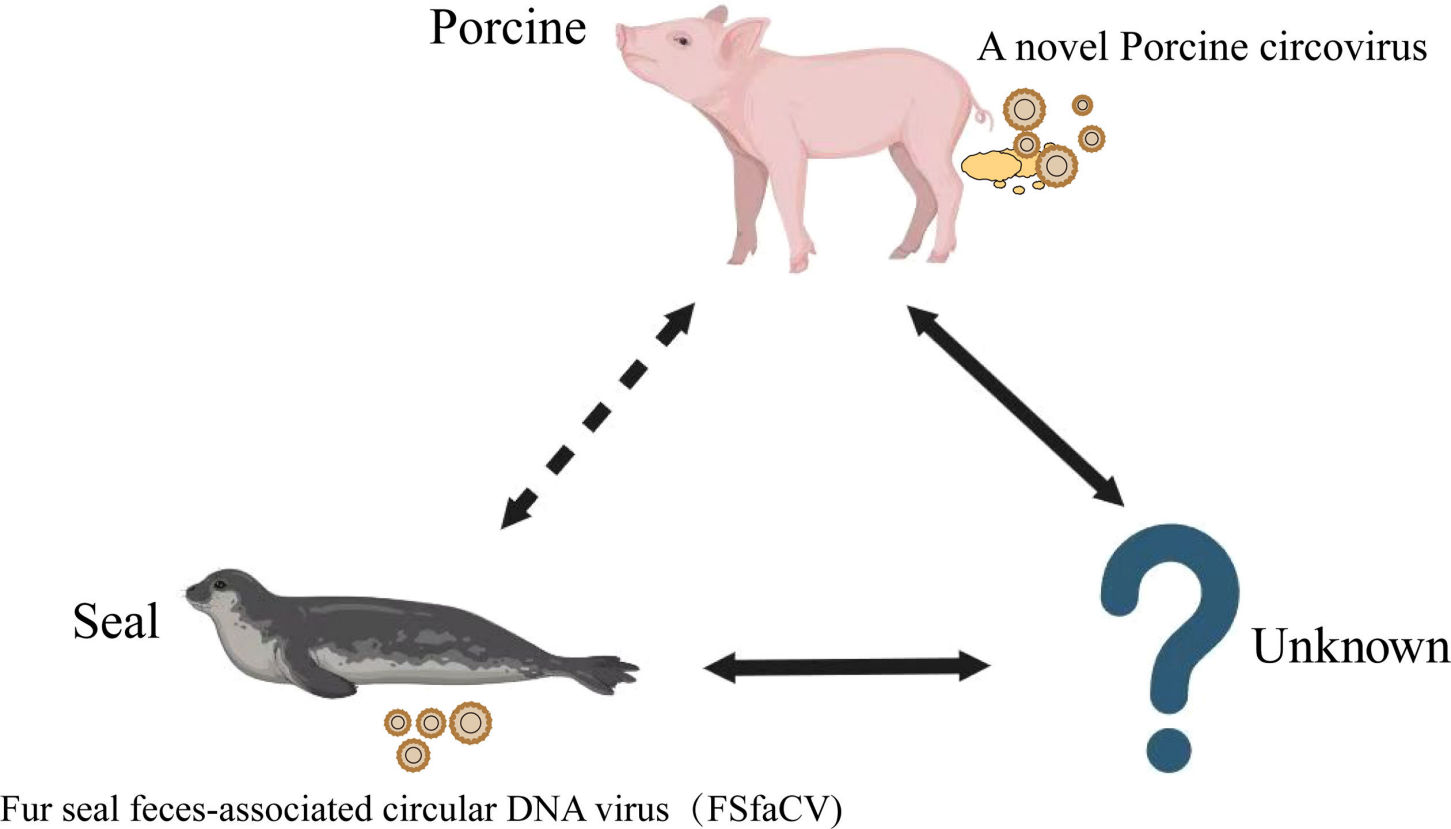
Diagram of infected animals with PCV5.

In this work, a novel single circular DNA virus identified from piglets is highly homologous to FSfaCV. The classification of CRESS DNA viruses primarily relies on Rep. The biochemical results about Rep of the virus further indicate that it can be classified into CRESS DNA viruses. Sequence alignment analysis revealed significant homology between PCV5 and FSfaCV. However, given the substantial difference in their primary host species, the study of FSfaCV is limited, and its prevalence is unknown, and the virus is closely related to PCVAD and is widely prevalent in pigs, which indicates that it may be an important pathogen; thus, the circovirus was named as PCV5. This nomenclature reflects the potential for distinct viral tropism between these two viruses. Furthermore, we cannot exclude the possibility of a cross-species transmission event as the origin of PCV5 (Fig. 8). Future studies will aim to provide further experimental evidence to elucidate the evolutionary origin of PCV5. We observed a close relationship between PCV5 and the severity of infectious disease. We then established an *in vitro* culture system for PCV5. Subsequently, PCV5 was successfully purified through sucrose gradient centrifugation, and morphological identification of the purified PCV5 revealed virus particles with a diameter of approximately 20 nm. We tried to establish an animal disease model in mice challenged with PCV5 (Fig. S6). However, the mice did not show any signs of infection, and PCV5 was not detected in multiple organs of the mice (Table S4), and all mice inoculated with PCV5 were alive during the observation period. These results indicated that PCV5 is not suitable for establishing a disease model in mice.

While much of the viral community on Earth remains largely unknown, there may be connections between some unknown human or animal diseases and CRESS DNA viruses. It is uncertain how close virologists are to uncovering the full extent of the diversity of CRESS DNA viruses. However, recent global efforts have more than doubled the number of CRESS DNA viruses in GenBank over the past decade (1, 2). There is no need to add new strain sequences to our understanding of the diversity of CRESS DNA viruses. These abundant sequences of novel CRESS DNA viruses come from a wide range of hosts and environments. The CRESS DNA viruses, in particular, have several characteristics that support their efficient transmission and spread, such as the ability to cross the placental barrier and blood-brain barrier, high viral copy numbers, and the stability of their circular DNA genomes (5, 6, 8, 12).

The researchers have better defined the diversity and prevalence of CRESS DNA viruses. The three longest-established families of CRESS DNA viruses include well-studied pathogens of animals and plants, although not all members of these families can cause disease in their hosts. The viruses of *Geminiviridae* and *Nanoviridae* infect plants, while the viruses of *Circoviridae* infect vertebrates (birds and mammals) and invertebrates (1). However, for other types of CRESS DNA viruses, *in vitro* propagation systems have not yet been established. The disease model and commercial vaccines for PCV2 have been widely accepted (5). However, other CRESS DNA viruses still face the challenge of Koch’s postulates. Therefore, many studies focus on the level of gene molecular analysis, concluding that a certain CRESS DNA virus is closely associated with a particular disease rather than establishing a definitive cause-and-effect relationship. This ongoing discussion has led to the debate surrounding the pathogenicity of CRESS DNA viruses. Of course, many researchers have also detected viral antigens in samples from diseased tissues, further confirming the close connection to the disease (8, 13–15).

The logic of viral evolution is defined by the key biological features of viruses, namely their obligate intracellular parasitic lifestyle (26). This lifestyle provides viruses with ample opportunities to directly exploit and manipulate the host’s cellular machinery, but it also necessitates overcoming the host’s defense systems (27). Viral evolution appears to be seeded by a set of core proteins involved in genome replication (Cap and Rep), which seem to have ancestral origins dating back to pre-cellular stages of evolution (26–28). Generally, the larger the viral genome of the PCV5, the more auxiliary functions it encodes. The growth of viral genomes is a result of acquiring various auxiliary genes that enhance the adaptability or operational autonomy of the virus, although they are largely redundant with the functions of the host cell (27). The evolution of larger genomes is determined by improved infection efficiency, broader host range, potentially higher attachment success rates, and reduced decay rates—characteristics that are particularly important in resource-limited environments with low host population densities (27). CRESS DNA viruses serve as excellent models for studying biological efficiency, viral replication, and assembly in the natural process of evolution (3). The CRESS DNA virus is often composed of a single Cap, making virus particles (18–23). The Rep protein, on the other hand, has an N-terminal endonuclease domain and a C-terminal ATPase and helicase domain (3, 16, 29).

Although PCV1 is classified as a non-pathogenic agent, PCV2, PCV3, and PCV4 significantly affect the immune system of pigs and induce severe diseases (5, 8, 9, 30). Some clinical symptoms caused by these viruses are collectively referred to as PCVAD (5). Over the past 20 years, PCV2 has been recognized as the primary cause of PCVAD, as its infection in pigs severely compromises the immune system, leading to immunosuppression and lymphatic system failure (5). PCV2 primarily proliferates in lymph nodes, inducing apoptosis in immune cells, which further weakens the immunity of infected pigs (5). Evidence indicates that PCV3 can lead to some pathological symptoms, including post-weaning multisystemic wasting syndrome (PDNS), diarrhea, reproductive disorders, respiratory, systemic inflammatory diseases, and central nervous system signs (31, 32).

In this article, we highlight that PCV5 is widespread in Southern China. To investigate the prevalence of PCV5 in healthy swine populations, surveillance studies were also conducted. We found PCV5 infection in clinically normal pigs, albeit with low viral titers and a low prevalence rate (data not shown). These findings suggest that PCV5 can establish subclinical infections in immunocompetent herds. Notably, during disease outbreaks on farms, PCV5 prevalence significantly increased. This pattern is consistent with potential latent infection, where viral reactivation may be facilitated by co-infection with other pathogens. Such synergistic interactions likely enhance PCV5 replication and pathogenesis, though experimental validation of this co-infection hypothesis requires further investigation. Regarding potential co-infection between PCV5 and PCV2/PCV3, a systematic analysis was not performed in this study. This knowledge gap stems from methodological differences: PCV5 was primarily detected in fecal samples, whereas PCV2/PCV3 screening relies predominantly on blood-based assays. Future studies will implement concurrent sampling of matched fecal and blood specimens to elucidate co-infection dynamics.

It is generally considered pathogenic and is associated with a variety of pathological symptoms similar to those caused by PCV2, including porcine diarrheal disease, respiratory disorders, and reproductive failure. Therefore, this novel CRESS DNA virus merits further investigation to clarify its significance and role in PCVAD.

## MATERIALS AND METHODS

### Samples and clinical background

In early 2022, the South China Agricultural University National Engineering Center for Swine Breeding Industry received clinical samples of different organs from approximately 12 piglets (5–20 kg) with varying degrees of respiratory and diarrheal disease. These piglets come from different litters. This farm has about 1,000 sows, and there is only a high mortality rate for farrowing piglets and nursery pigs. And the health status indicators of pigs are poor, always accompanied by respiratory diseases and diarrheal diseases. Pig farmers have not found the cause, and there are no good measures to change this phenomenon. Only PCV5 was positive, and quantitative real-time PCR (qRT-PCR) was developed to detect the loading titer of PCV5. Subsequently, we initiated a surveillance program on the epidemiology and pathogenicity of PCV5. The clinical samples, including respiratory, diarrheal, and reproductive failure diseases of pigs, were obtained from 70 swine farms in different regions of China from October 2021 to June 2023 and stored at −80°C.

### DNA/RNA extraction of viruses

Clinical samples were grounded using PBS at 4°C, and samples were repeatedly freeze-thawed three times. Subsequently, the viral DNA/RNA of the sample is extracted with RaPure Viral RNA/DNA Kit (Magen, R4410-3, China).

### PCR array

Microorganisms and viruses related to intestinal diseases, respiratory diseases, and reproductive disorders of pigs were detected by laboratory-preserved test methods (10, 33). Multiple publicly available CRESS-DNA virus sequences were retrieved from genetic databases and subjected to sequence alignment. This analysis revealed conserved motifs within the Rep gene, which were subsequently used to design primers for amplification. Following PCR amplification using these conserved-region primers, the resulting nucleic acid products were sequenced and subjected to phylogenetic tree construction. Primers used to obtain PCV5 genome sequences were designed (Table S5).

### Real-time PCR array

To further investigate tissue tropism of PCV5 in piglets suffering respiratory and diarrheal disease, a SYBR green qRT-PCR targeting the conservative regions of PCV5 was developed based on the obtained PCV5 virus strains. And the designed primers were tested and showed good sensitivity, specificity, and reproducibility. Detailed information about gene primers used in the PCR and qRT-PCR is listed in Table S5.

### Phylogenetic analysis

The complete gene sequences of PCV5 viruses obtained in this article have been uploaded to GenBank with the accession numbers (Table S1). The virus genome was assembled using SnapGene. All arrangements were further aligned using the ClustalW alignment method in MegAlign (Lasergene). The phylogenetic tree was built using the maximum likelihood method and 1,000 bootstrap replicates with MEGA software. The amino acid sequence of the gene was compared by DNAman and SDT V1.2 software.

### Alpha-Fold2 predicts the structure of open reading frames

The complete three-dimensional structure of open reading frames obtained after whole-genome analysis was predicted using the artificial intelligence prediction software Alpha-Fold2. Alpha-Fold2 software, developed by DeepMind, was trained using artificial intelligence algorithms based on a data set of over 190,000 protein structures from the Protein Data Bank (PDB) (https://www.rcsb.org). This algorithm accurately predicts the three-dimensional structure of a protein based on its input amino acid sequence. On our local computing server, we input the complete amino acid sequence of the open reading frame and execute the Alpha-Fold2 command, which outputs five predicted structures. Based on the probability distribution, we selected the highest probability ranked_0.pdb file for further structural analysis. The analysis and domain delineation of the structure were performed using the PyMOL software.

### Cells and reagents

Dulbecco’s modified Eagle medium (Gibco-BRL) containing 10% fetal bovine serum (Sorfa Life Science) and 1% penicillin-streptomycin (Gibco, Thermo Scientific) was used for maintaining MDCC-MSB1 cells, PK15 cells, and HeLa cells (ATCC CRL-11268), which were incubated at 37°C in 5% CO_2_.

### Virus purification

The cell medium was clarified by low-speed centrifugation (4,000 rpm) for 10 min at 4°C to remove the cell debris. For the discontinuous sucrose gradient centrifugation, the supernatant was purified with a discontinuous 30%–50% (wt/vol in PBS) sucrose gradient and centrifuged with an SW32.1 rotor (Beckman) at 100,000 × *g* for 3 h at 4°C. Fractions with PCV5 particles were collected. For the continuous sucrose gradient centrifugation, the continuous 10% to 50% (w/v in PBS) linear sucrose gradient was made using a Gradient Master (BioComp Instruments Inc., Canada). The concentrated virus was then placed on a 10%–50% linear sucrose gradient and centrifuged with an SW32.1 rotor (Beckman) at 140,000 × *g* for 14 h at 4°C. Fractions with PCV5 particles were collected. The purified PCV5 particles were then examined by western blot and imaged with negative staining EM.

### Negative stain

For examination by negative staining, an aliquot of 4 μL of purified virus was applied to freshly glow-discharged carbon-coated copper grids (Zhong Jing Ke Yi Corp., China). After 1 min, the excess liquid was removed using a filter paper. The grid was then stained with 2% uranyl acetate for 40 s and removed using a filter paper. All samples were examined on a Tecnai T12 electron microscope (FEI) operated at an acceleration voltage of 120 kV. Images were recorded using a CCD camera (Eagle, FEI).

### Construction of prokaryotic and eukaryotic plasmids

The full-length Cap and different truncated Cap constructs were cloned into a prokaryotic expression plasmid. However, only the Cap protein truncated by 63 amino acids could be efficiently expressed and purified in pET-28a, and it was able to assemble into virus-like particles.

The full-length Cap was fused to the C-terminus of pCMV plasmid with a 3×FLAG tag and to the C-terminus of pCMV plasmid with a Twin-STREP tag. The full-length Rep was fused to the N-terminus of pCMV plasmid with a 3×HA tag. Detailed information on the gene primers used can be found in Table S6.

### Expression and purification of recombinant proteins

Transform the successfully constructed plasmids into BL21(DE3) competent cells. Inoculate a single positive colony into 5 mL of Luria-Bertani broth (LB) medium containing the appropriate antibiotic and incubate overnight at 37°C with shaking. Then, transfer 5 mL of the overnight culture into 1,000 mL of LB medium supplemented with antibiotics and continue shaking at 37°C for 4–6 h until the OD_600_ reaches 0.6–0.8. Reduce the temperature to 16°C and add isopropyl β-D-1-thiogalactopyranoside (IPTG) to a final concentration of 0.2 mM, then continue shaking for 16–18 h to induce protein expression.

The total protein from the obtained bacterial culture was subjected to initial affinity nickel column purification, followed by ion exchange chromatography and size-exclusion chromatography, to obtain the desired protein with high purity and uniform oligomeric state.

### Indirect immunofluorescence assay

Indirect immunofluorescence assay allows for the visual observation of the expression and distribution of the target protein in cells. It involves the use of specific antibodies (primary antibodies) that bind to the target protein, followed by the binding of fluorescently labeled secondary antibodies to the primary antibodies, generating fluorescence. The samples are then observed under a fluorescence microscope.

### Preparation of polyclonal antibodies against Cap protein and Western blotting

The recombinant Cap virus-like particle protein is administered to immunize rabbits four times with an interval of approximately 3 weeks, with each immunization consisting of 1 mg of Cap protein along with an adjuvant. After the immunizations, the serum from the rabbits is collected, which contains polyclonal antibodies against Cap protein. For the Western blotting assay, clinical samples are lysed, and the obtained rabbit serum is used as the primary antibody to detect the presence of viral proteins in the tissues.

### Development of a recombinant PCV5 Cap VLPs ELISA

The 30 serum samples obtained from a specific pathogen-free herd that tested PCV5 negative by qPCR were used as negative controls and had an average absorbance of 0.167. The cutoff value differentiating negative and positive serum samples was determined as 3 standard deviations above the mean of the negative controls (0.359). The protocol of ELISA was similar to assays previously described (34), by using 0.25 µg/mL purified PCV5 Cap VLPs to coat the wells.

## SUPPLEMENTAL MATERIAL

Supplemental material spectrum.02299-25-s0001.pdf Fig. S1 to S6; Tables S1 to S6.

ASM does not own the copyrights to Supplemental Material that may be linked to, or accessed through, an article. The authors have granted ASM a non-exclusive, world-wide license to publish the Supplemental Material files. Please contact the corresponding author directly for reuse.
