# Supplementary material for: Identification and biochemical characterization of a novel porcine circovirus associated with porcine respiratory and diarrheal diseases
Source: Microbiol Spectr. 2025 Oct 8;13(11):e02299-25. doi: 10.1128/spectrum.02299-25 (PMC12584716; doi:10.1128/spectrum.02299-25)
Supplement: Supplemental material — Fig. S1 to S6; Tables S1 to S6. [file spectrum.02299-25-s0001.pdf]

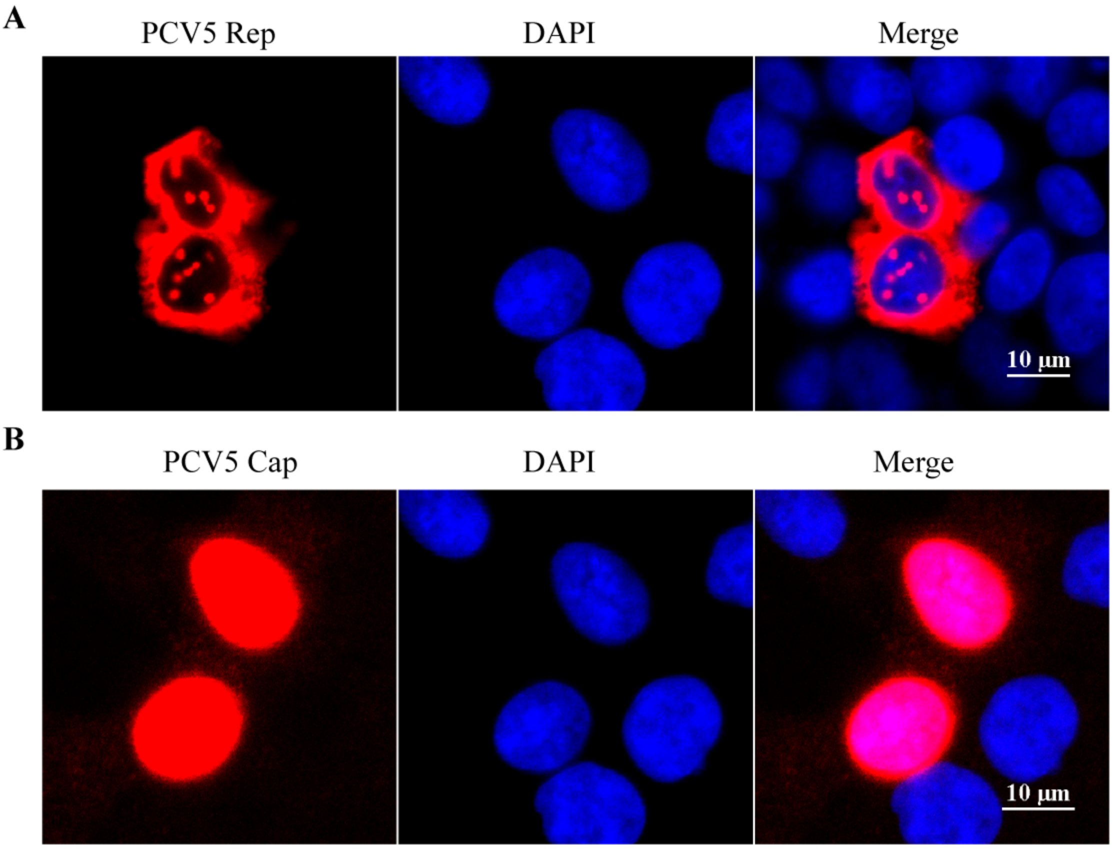

Figure S1. Subcellular localization of PCV5 Cap and Rep. Hela cells were transfected with 600 ng of PCMV-FLAG-Cap, PCMV-HA-Rep or empty plasmid. The cells were then fixed, and IFA was used to detect PCV5 Cap (red) and PCV5 Rep (red). DAPI (blue) was used to stain the cellular nuclei, and a confocal microscope was used to obtain the fluorescent images.

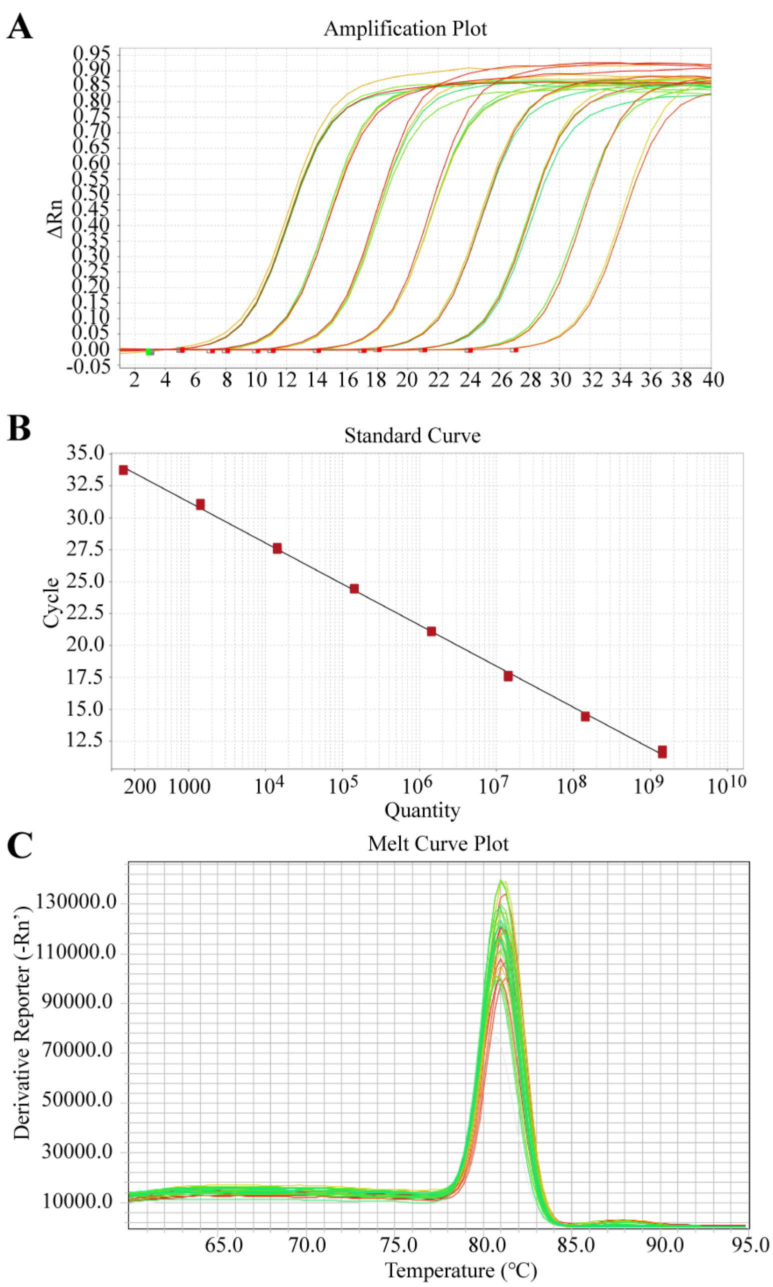

Figure S2. Real-time qPCR detection of PCV5. an amplification plot, a standard curve and melting curve analysis. The plot of amplification ranging from 1×10^2 copies/μL to 1×10^9 copies/μL (A). Standard Curve represents the copy number with the corresponding Ct values, which ranges from 1×10^2 copies/μL to 1×10^9 copies/μL (B). The melting curve indicates a melting peak at 81 ± 0.5◦C with single and of good quality (C).

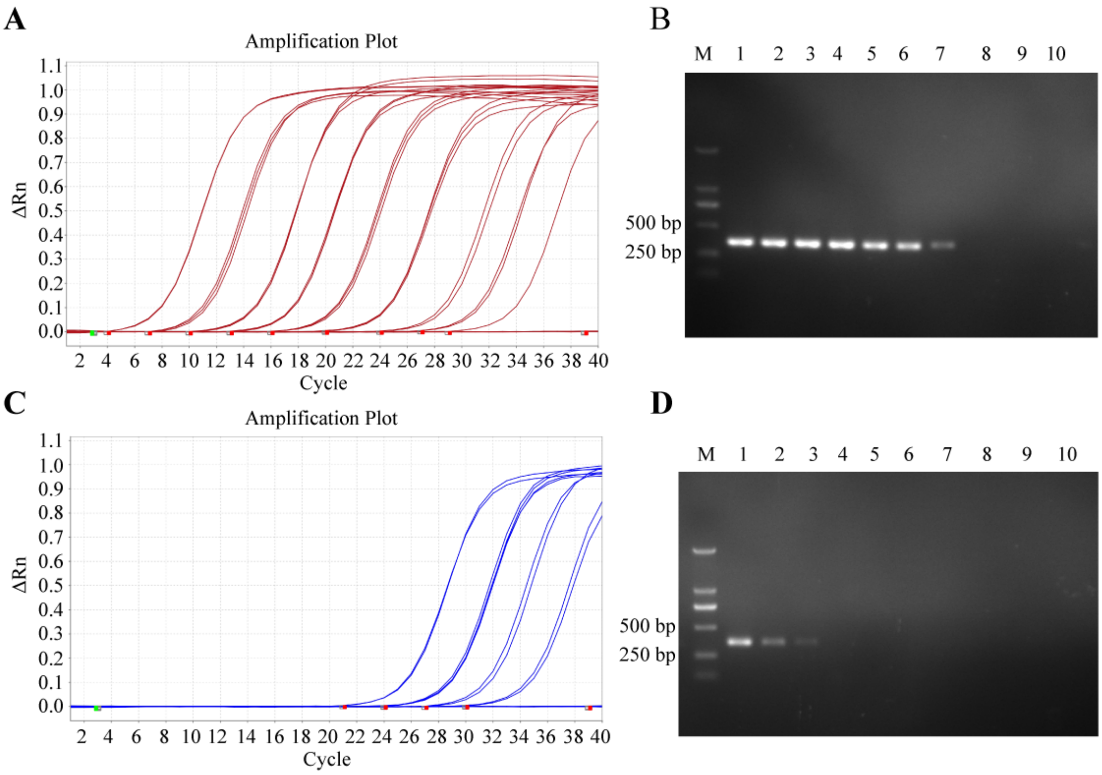

Figure S3. Sensitivity analysis of Real-time qPCR. The constructed PCMV-HA (N)-PCV5-Rep plasmid and the PCV5 nucleic acid were diluted 10 times for detection. The PCR and real-time qPCR were used to detect the diluted plasmid (A, B) and the PCV5 nucleic acid (C, D), respectively; 1, 2, 3, 4, 5, 6,7, 8 and 9 represent different dilutions.

A

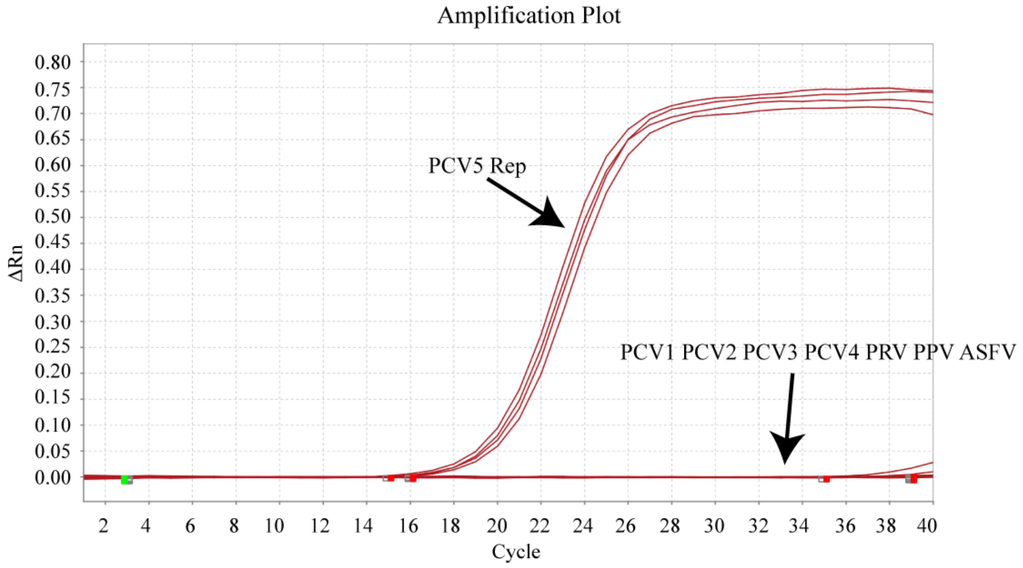

B

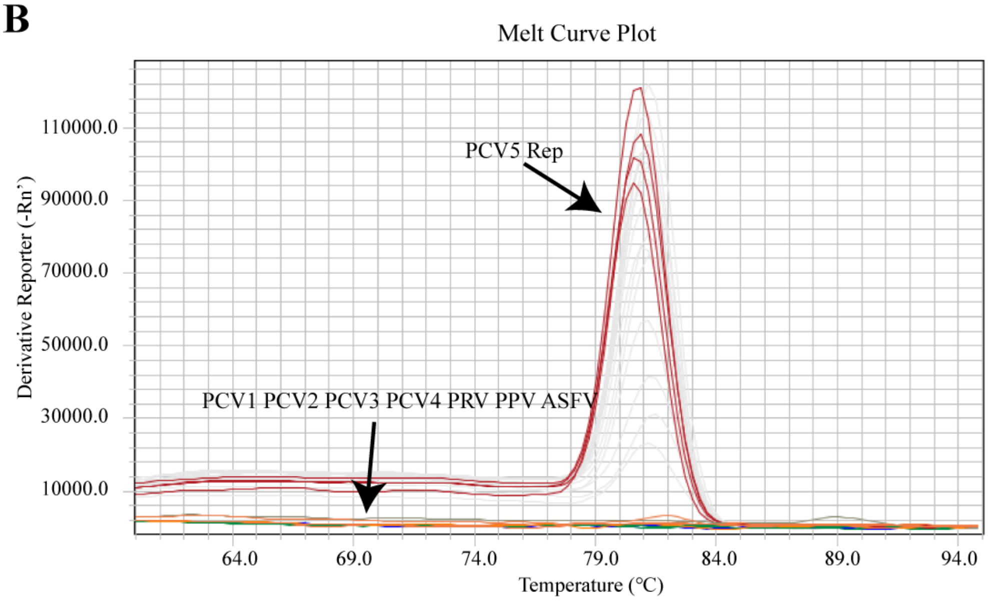

Figure S4. Specificity analysis of Real-time qPCR. The DNA of PCV1, PCV2, PCV3, PCV4, PPV, PRV, ASFV, et were used as templates for Real-time qPCR. The amplification plot (A) and melting curve analysis (B) was shown in the figures.

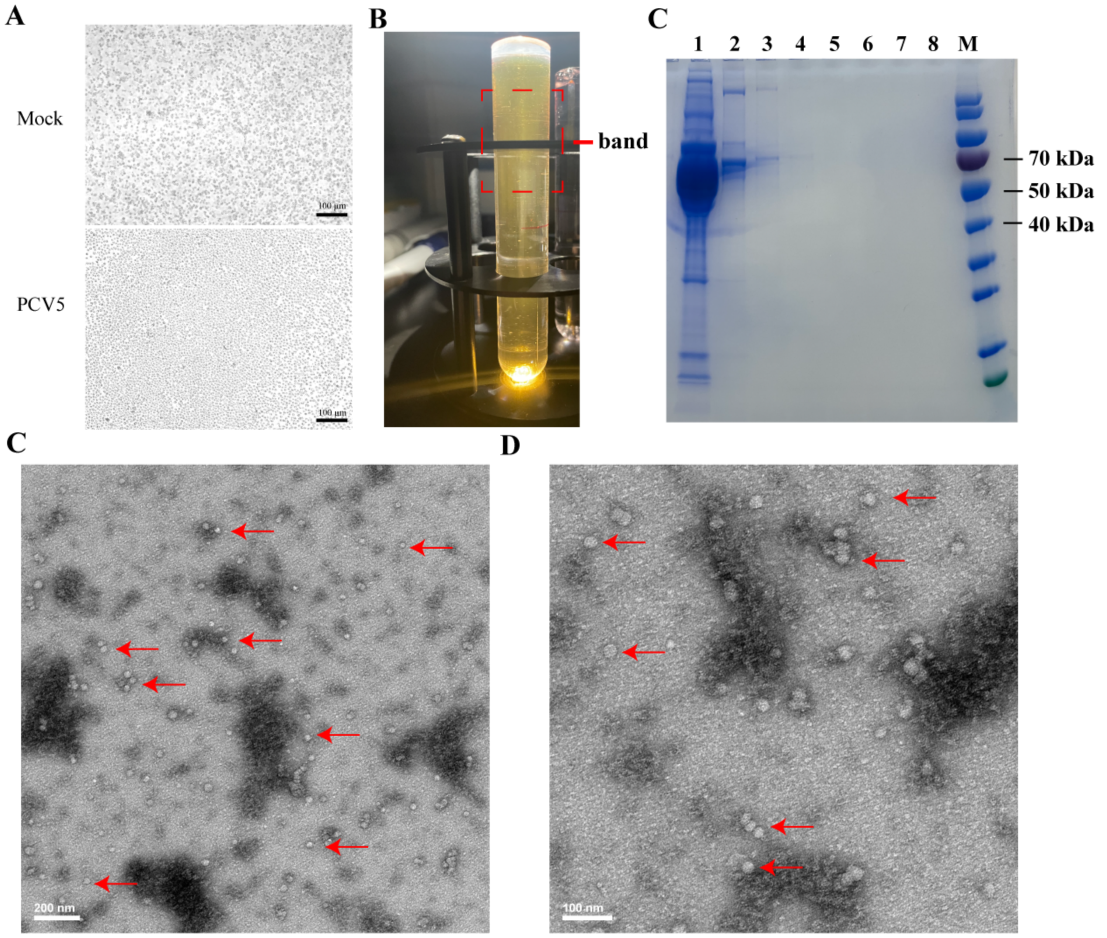

Figure S5. In vitro propagation and identification of PCV5 virus. (A)MDCC-MSB1 cells infected with PCV5 showing no Cytopathic effects (CPE). (B) Purification of PCV5 using continuous 10% to 50% (w/v in PBS) linea sucrose gradient centrifugation. (C) SDS-PAGE analysis of fractions from discontinuous sucrose gradient centrifugation. Lane1 to lane 7 represent the band (500 μL from top to bottom). (D) Negative stain microscope detection of purified PCV5 virus. The scale bar is 200 nm. (F) Negative stain microscope detection of purified PCV5 virus. The scale bar is 100 nm.

A

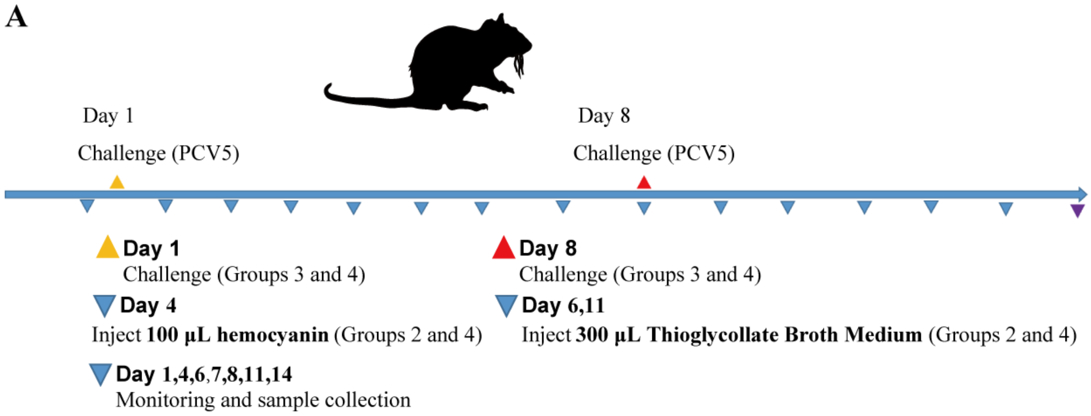

B

Group (Animal number)	Group1 (5)	Group2 (5)	Group3 (5)	Group4 (5)
**Challenge**	**PBS(300 µL)**	**PBS(300 µL)**	**PCV5(300 µL)**	**PCV5(300 µL)**
**Hemocyanin and Thioglycollate Broth**		√		√

Figure S6. An animal disease model using mice challenged with PCV5 (A) The 5 mice in four groups were intramuscularly injected with PCV5, and the first group were taken as control. The mice were monitored and the body temperature and clinical symptoms were recorded. (B) Grouping of all mice and experimental conditions.

Table S1. List of the PCV5 strain

Farm	The name of PCV5 strain	the full genomes of virus (nt)	Stem loop	Genbank number
A	PCV5_2904_CY	2904	gacataaggggcgcagctctagtattacccttatgtc	OR842903.1

Note: The name of the circovirus strain in this article is tentative. In this paper, a virus sequence was uploaded first, which was temporarily named PCV5, and the remaining 11 full gene sequences were waiting to be uploaded to the GenBank.

Table S2. Antigen concentration and serum concentration (OD_{450} nm value)

Dilution of sera		Antigen at different concentration (μg/mL)					
		0.1	0.25	0.5	1	2	4
1:25	P	2.504±0.015	2.474±0.026	2.641±0.023	2.623±0.036	2.586±0.055	2.635±0.071
	N	0.06±0.005	0.079±0.007	0.103±0.004	0.172±0.012	0.173±0.003	0.218±0.009
1:50	P	2.271±0.020	2.524±0.025	2.619±0.019	2.764±0.035	2.651±0.039	2.703±0.033
	N	0.059±0.017	0.071±0.008	0.081±0.003	0.103±0.010	0.236±0.012	0.161±0.021
1:100	P	2.336±0.028	2.456±0.019	2.55±0.037	2.828±0.051	2.829±0.042	2.754±0.037
	N	0.055	0.056±0.013	0.068±0.010	0.079±0.018	0.12±0.012	0.142±0.007
1:200	P	2.133±0.017	2.041±0.029	2.473±0.031	2.59±0.026	2.404±0.039	2.588±0.045
	N	0.054±0.012	0.059±0.003	0.076±0.012	0.08±0.025	0.062±0.019	0.065±0.032

Table S3. A PCV5 ELISA detect OD_{450} nm values of other pathogens in pigs

Other pathogens	OD_{450} nm
PCV2	0.071±0.035
PCV3	0.092±0.048
PPV	0.032±0.071
PRV	0.112±0.023
PRRSV	0.052±0.042
PEDV	0.052±0.042
PCV5	1.221±0.057

Table S4. PCV5 CT values of organs in different groups of mice on dpi 14

Group / Part	Group1 (5)	Group2 (5)	Group3 (5)	Group4 (5)
Heart	-	-	-	-
Liver	-	-	-	-
Spleen	-	-	-	33.541
Lung	-	-	-	-
Nephridium	-	-	-	-
Duodenum	-	-	-	-
Jejunum	-	-	-	-
Ileum	-	-	-	-

Table S5. qPCR and PCR primers used in determine PCV5

Name	Sequence of primers (5’ → 3’)	Use
2800-1-F	TTTAAGTATGGATTTATGAAGTGTTGT	
1315-1-R	AAGCACCATAACCCTTACCAAGTTG	Genome sequence
1020-2-F	TATTGGATGGGTCCATGTATCCGTCT	
2350-2-R	GTCAGATATTTGTCAATCGGAAAAC	Genome sequence
2000-3-F	TACTTCTCAAGTCCACTAGAGTATCTT	
230-3-R	ACCACTTACAGTTAAACCTGAGGACTT	Genome sequence
RT-F	ACGGTGCTAGCAAAGTGTGA	PCV5 forward primer
RT-R	GCCAACTACCAAGCCGAAGA	PCV5 reverse primer

Table S6. Primers used for plasmid construction in this study

Name	Sequence of primers （5’ → 3’）
PCMV-HA-N-R	AATTCGGGCCTCCATGGC
PCMV-HA-N-F	CTCGAGGTACCGCGGCC
HA-REP-F	CCATGGAGGCCCGAATTATGCCTGCCGTACGTGTGAAA
HA-Rep-R	CCGCGGTACCTCGAGTTAATCGATATCAAGAATAGAATCATCT GGTTGT
CAP-FLAG-F	CTCGGATCCGCCACCATGTCACCTGCGCCTG
CAP-FLAG-R	CTTTGTAGTCCTCGAGTGCCTGATAATCAGGGAAGATAGGG
PCMV-FLAG(C)-F	GGTGGCGGATCCGAG
PCMV-FLAG(C)-R	CTCGAGGACTACAAAGACCATGAC
PCV5 Cap Δ63-F	TGTATTTTCAGGGATCCGGTCTGACCGTTAGCGGTA
PCV5 Cap Δ63-R	CCGCAAGCTTGTCGACTTACGCCTGGTAATCCGGGAAGA
pET-28a-F	GGATCCCTGAAAATACAGGTTTTCG
pET-28a-R	GTCGACAAGCTTGCGGC
